# Detection, Location and Grasping Objects Using a Stereo Sensor on UAV in Outdoor Environments

**DOI:** 10.3390/s17010103

**Published:** 2017-01-07

**Authors:** Pablo Ramon Soria, Begoña C. Arrue, Anibal Ollero

**Affiliations:** Robotics, Vision and Control Group, University of Seville, Camino de los Descubrimientos, s/n, Seville 41092, Spain; barrue@us.es (B.C.A.); aollero@us.es (A.O.)

**Keywords:** UAV, grasping, outdoors

## Abstract

The article presents a vision system for the autonomous grasping of objects with Unmanned Aerial Vehicles (UAVs) in real time. Giving UAVs the capability to manipulate objects vastly extends their applications, as they are capable of accessing places that are difficult to reach or even unreachable for human beings. This work is focused on the grasping of known objects based on feature models. The system runs in an on-board computer on a UAV equipped with a stereo camera and a robotic arm. The algorithm learns a feature-based model in an offline stage, then it is used online for detection of the targeted object and estimation of its position. This feature-based model was proved to be robust to both occlusions and the presence of outliers. The use of stereo cameras improves the learning stage, providing 3D information and helping to filter features in the online stage. An experimental system was derived using a rotary-wing UAV and a small manipulator for final proof of concept. The robotic arm is designed with three degrees of freedom and is lightweight due to payload limitations of the UAV. The system has been validated with different objects, both indoors and outdoors.

## 1. Introduction

The use of unmanned aerial vehicles (UAVs) is becoming increasingly popular; not only for military purposes, but also in many other fields, from wildlife and atmospheric research to disaster relief and sports photography [1]. Applications in agriculture and industrial environments are currently being exploited, for example, in inspection and maintenance. They can be used as a fast and safe response in a critical situation against disasters, plagues, or working in dangerous or inaccessible places.

Some important incidents—such as the nuclear disaster in Fukushima in 2011—also clearly illustrated the problem that UAVs are today nearly exclusively used as flying sensors. UAVs are equipped with different types of sensors providing situational awareness, but are so far not equipped with any type of actuators, unlike many ground-based mobile robots. Unfortunately, flying manipulation comes with many unsolved problems. A suitable manipulator that can be attached to a small UAV must also be small and lightweight.

The work presented in this paper is focused on the development of an on-board perception system for autonomous object manipulation using UAVs. The objective is to provide the aerial robot with capabilities to perform inspection and maintenance tasks (which imply manipulation) that can be dangerous for human operators or in places that are difficult to reach. Sending UAVs will minimize the risks and increase the response speed and automation of operations.

Currently, perceiving the environment remains a challenging task. The robot needs to recognize the targeted objects and minimize false positives. Particularly, in aerial vehicles, a wrong detection can result in disastrous consequences for the platform. The use of different machine learning algorithms for object detection has rapidly expanded. Algorithms such as Support Vector Machines (SVM) and Neural Networks (NN) have been used successfully for this task.

For grasping objects, it is also necessary to analyze the 3D information of the object. Estimating its pose accurately is needed so that a manipulator can grasp it. The use of a monocular camera requires the recovery of the 3D information. Utilizing depth sensors, stereo cameras, or lasers can simplify the task, as they directly provide this information.

Once the object is found and located relative to the robot, it is necessary to analyze how to perform the grasp. Usually, the object is defined as a mesh or a primitive shapes (spheres, cylinders, etc). This information can be provided by depth sensors, stereo cameras, or lasers, producing point clouds and processing them. Grasping information is generated using the full 3D shape with approaches like force-closure and/or quality metrics [2]. However, the object might be occluded in real applications, in addition to the fact that these kinds of methods can be time consuming and may be impractical if the object is fully known.

Our work focuses on the task of object detection and pose estimation for real-time tasks on UAVs for grasping objects. We only consider objects that contain textured surfaces and which are graspable by the UAV (for example, the algorithm will not be able to learn a plain white folder, and the UAV the manipulator on the UAV cannot grasp objects larger than the gripper) The algorithm is based on the work presented in [3,4], but varies in the use of stereo cameras to improve the learning process (as they provide 3D information) and introduces improved feature filtering. Furthermore, as proof of concept, the UAV has been equipped with a three degrees of freedom (DOF) arm with a gripper as end-effector.

The remainder of this article is structured as follows. In Section 2 we describe the related work. Section 3 is divided in three subsections: feature extraction and filtering, creation of object model, and how to find the object in new scenes. Section 4 shows the validation tests we performed, and the final section presents the conclusions and future work.

## 2. Related Work

As mentioned before, for the development of an on-board perception system for autonomous object manipulation using UAVs, the robot needs to detect the targeted tools to be grasped for the task. This process can be split into three parts. The first part involves the detection and recognition of the object, the second is the estimation of its position (and orientation), and the third is the grasp analysis. Object detection is a wide area of research: there exist several algorithms depending on the prior knowledge, the environment, and also on the sensor used. Some authors use machine learning algorithms as neural networks [5], or classifiers such as Support Vector Machine (SVM) [6] using a bag-of-words model. However, for grasping objects, it is also necessary to estimate the position of the object and grasping points. Authors in [7,8,9] used depth sensors such as Kinect to model the object and segment it using the depth information. Depth information is very useful for the estimation of object position, but the sensors usually have limitations in outdoor environments, as they use IR-structural light. Other authors use monocular cameras aided with geometrical models of the objects, as in [10]. Authors in [3,4] showed how to describe objects using image features to recognize and estimate its position accurately while being robust to occlusions. Finally, it is necessary to analyze how to grasp the object. Authors in [2] show a complete survey about quality metrics for comparing different grasps using the available geometrical information of the object to generate virtual grasps. This geometrical information can be provided by depth sensors [11], cameras [12], or haptic sensors [13]. In [14], authors used SVM to estimate the quality of the grasp.

References [15,16,17,18] studied the grasping of objects using UAVs, paying particular attention to the mechanical and control aspects of the grasp. In general, for grasping with aerial robots, the authors assume that the position of the object is known, or they use simple markers such as color or tags [19,20,21]. In [22], authors studied the dynamics, control, and visual servoing of an aerial vehicle for grasping inspired by animal movements. Nevertheless, they assume that the object is a cylinder with known radius. Conversely, in this article, the proposed algorithm allows the robot to robustly detect more complex objects. In this work, it is assumed that the UAV has an internal control that is sufficient for movement and manipulation. The arm is lightweight, and the movements are slow enough that the control system is able to stabilize the robot.

In a previous work, the authors [23] developed a system with a low-cost stereo camera for object detection and location. In this system, a local map is created to localize a list of object candidates and the relative position of the UAV on the map. Once the candidates are located, their volume is projected into the images, and a machine learning algorithm is run to recognize the object category. This was called a bottom-up approach which firstly detects that there is something in the environment, and then categorizes the object.

**Figure 1:**
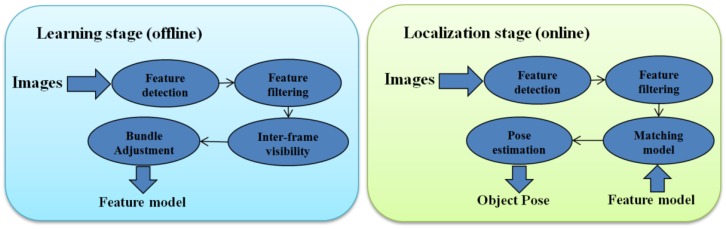
Pipeline of both stages of the algorithm.

In the present paper, a top-down approach is used. The kind of objects that are to be grasped were known, and it was possible to generate a model for each one. In an offline stage, the algorithm learned the model using visual features. Afterwards, in the online stage, the model was used to seek and locate the object (see Figure 1). A small arm was 3D printed to test the grasping results, and everything ran on an on-board Intel NUC computer [24]. Finally, for image acquisition, the UAV was equipped with a commercial stereo camera called ZED [25]. Through its SDK, this camera can be used to compute depth maps using CUDA (NVIDIA parallel computing platform and API for Graphic Processor Units), but this software has range limitations and cannot be used for distances shorter than one meter. Nonetheless, the built-in cameras have high quality, the auto-focus and auto-exposure are remarkable, their construction is stiff, and they provide a versatile combination between resolution and frames per second (FPS). Moreover, the baseline of the camera (120 mm) is good for a reconstruction on relatively short distances (within 20–100 cm). For these reasons, this camera was chosen for this project. The object detection and pose estimation algorithm was tested on-board, and it was shown to be robust to vibrations, occlusions, and illumination changes. However, for security reasons, the robot was hanging on a structure while testing the grasping algorithm.

## 3. Object Detection and Pose Estimation

This algorithm is divided into two stages: the learning stage (or offline stage), in which the model of the object is created, and the localization stage (or online stage), in which the object is detected and its position is estimated. Figure 1 summarizes the pipeline of the algorithm in both the modeling and localization stages.

### 3.1. Feature Extraction and Filtering Using Stereo Cameras

Image features have been widely used and studied. SIFT (Scale-Invariant Feature Transform) [26] is a well-known detector and descriptor, and it has been proven to be robust to scales, rotations, and translations on images. However, it has a high computational time. Authors in [27,28] proposed some optimization to speed it up, but it is sometimes still not fast enough, or some losses of information are caused by their approximation. Vision algorithms for UAVs have a strong speed requirement, as they need a faster response for the control loop than ground robots. Several new feature detectors and descriptors have been developed, such as SURF (speeded up robust features) [29], ORB (Oriented FAST and Rotated BRIEF) [30], DAISY (an efficient dense descriptor applied for wide baseline stereo) [31], BRIEF (Binary Robust Independent Elementary Features) [32], FAST (features from accelerated segment test) [33], and more. These methods have been designed according to different objectives, such as being faster or more robust.

The performance of the image feature detection and matching for the model creation and object position estimation depends on the combination of the detector and descriptor chosen. Nevertheless, for both the object modeling and detection algorithms, the features are completely exchangeable. In Section 4, we show the processing time for the different combinations of detectors and descriptors.

**Figure 2:**
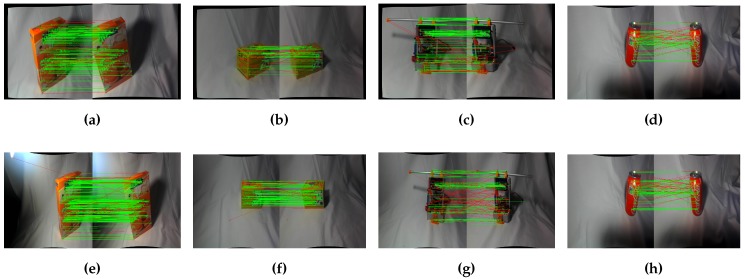
Filtering bad features using known stereo geometry. (**a**) Whoopies box 640 × 480; (**b**) Gena box 640 × 480; (**c**) Drilling tool 640 × 480; (**d**) Coke 640 × 480; (**e**) Whoopies box 1280 × 720; (**f**) Gena box 1280 × 720; (**g**) Drilling tool 1280 × 720; (**h**) Coke 1280 × 720.

As mentioned before, a ZED stereo camera was used for image acquisition. This particular camera has a wide-angle lens, so a rectification of the images is needed to properly detect and match the features. Initially, features are detected on each pair of images, then these features are matched with either a FLANN (Fast Library for Approximate Nearest Neighbors) [34]-based matcher or just a force brute matcher. The first advantage of using stereo images is to improve the filtering of matches, making it more robust to outliers. Despite this, it needs more process time, as features are computed in both images. The resulting inliers are assumed to be key features of the object. This makes our algorithm more robust to outliers, as they are rejected at the beginning of the process, so only the features that are more invariant are used. Figure 2 shows examples of feature filtering using stereo, as described in this paragraph.

### 3.2. Object Modeling

The learning stage (or offline stage) generates a model of an object from a set of images. This approach differs from [3,35] in the use of a stereo camera, automating the learning process (providing 3D information of the real-world scale) and improving the filtering of outliers. The process is summarized in the following points:

- Image rectification from camera calibration.
- Detection of features on both images and matching them. Use of stereo geometry and RANSAC (Random sample consensus) to filter outliers.
- Matching of sequentially filtered features.
- Performance of bundle adjustment to create a 3D model of the object and store the corresponding descriptors.
- Scale model of the object to its real size using stereo information.

The first stages of the object modeling are feature detection and filtering using the stereo system and its calibration following the procedure described in previous section. Once all the images are processed, and the corresponding set of cleaned features is obtained, a bundle adjustment (BA) of both features and camera positions is performed [36] in order to reconstruct the correct object shape. Further, the camera positions—where the images were taken—are obtained (however, this information is not used for the proposed method). We used the library cvSBA as implementation for the Sparse Bundle Adjustment (SBA). It is a wrapper of the SBA library [37] for use with OpenCV.

**Figure 3:**
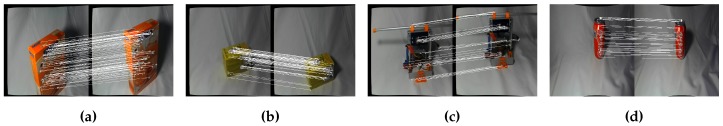
Sequential association of features to compute their inter-frame visibility. (**a**) Whoopies box 640 × 480; (**b**) Gena box 640 × 480; (**c**) Drilling tool 640 × 480; (**d**) Coke 1280 × 720. Then, following Algorithm 1, the visibility between non-sequential frames is computed.

In order to perform the BA, it is necessary to correlate the points within all the images. Only left projections are used for this step, since cvSBA does not allow users to establish custom restrictions between camera frames. Nonetheless, left projections contain enough information to reconstruct the object (the stereo information will be used to scale the object after the optimization, as described later). It is assumed that all of the pictures from the dataset are arranged as they were captured. Then, the features are matched sequentially to obtain the inter-frame visibility of the features. With this step, we obtained the relations in sequential frames (Figure 3 shows sequential matches of features for the creation of the inter-frame visibility matrix).

**Figure 4:**
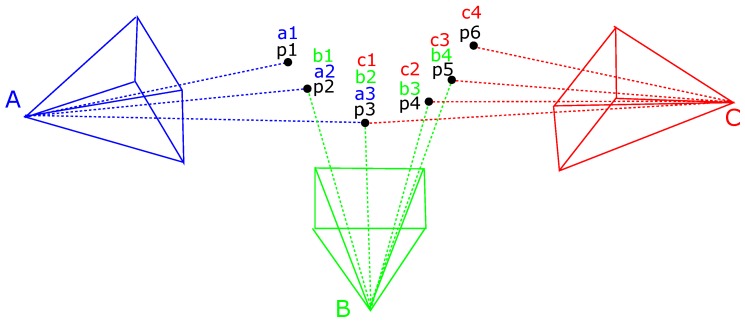
Diagram of elements in the Bundle Adjustment problem. A, B, and C represent the position of the camera from where the observations were taken. $p_{i}\forall i=1...6$ are six features in the space and $a_{i},b_{i},\;\mathrm{and}\;c_{i}$ are the features observed by each of the positions.

Nevertheless, it is necessary to obtain the remaining relations within all of the frames. Let us denote $P=\{p_{k}\;\forall \;k=1...K\}$ a vector in which each $p_{k}$ is a vector with the features on the frame *k*; *M* a matrix where each element $m_{ij}$ contains a vector with the matches between the frame *i* and the frame *j*. All the elements $m_{i(i+1)}$ are filled from the sequential match of frames. Then, the rest of the elements of $m_{ij}$ above the diagonal (i.e., j>i) can be filled using Algorithm 1. An improvement can be made in detecting loop closure; however, for most cases, this method is enough to reconstruct the object. A diagram of this visibility problem is shown in Figure 4.

**Algorithm 1** Correlate back matches  1:**for** offset=2,offset<K **do**  2: **for** i=0,i<K−offset **do**  3:  j=i+offset  4:  **for** $\mathrm{match}\;\mathrm{in}\;m_{i(j-1)}$ **do**  5:   **if** $\mathrm{match}\;\mathrm{is}\;\mathrm{visible}\;\mathrm{in}\;m_{(j-1)j}$ **then**  6:    add match in $m_{i}j$  7:   **end** **if**  8:  **end** **for**  9: **end** **for**10:**end** **for**



**Figure 5:**
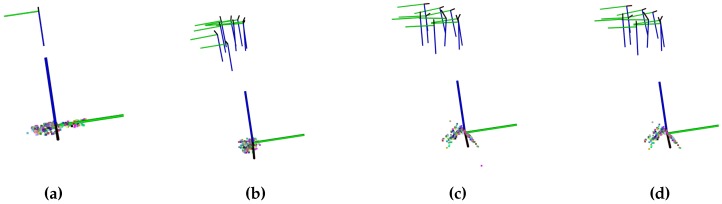
Different steps of the Bundle Adjustment optimization process. (**a**) Starting state; (**b**) First iteration; (**c**) Iteration 5; (**d**) Iteration 20.

The BA consists of a global optimization using Levenberg and Marquardt’s [38] algorithm to minimize the re-projection errors. Let there be a set of *N* 3D points, observed from *K* cameras (at $T_{k}$ position and $R_{k}$ orientation). Then, given the correlations between the projections of the 3D points into the cameras, an optimization is performed, minimizing the errors. Using the previously defined matrix *M* of matches between frames, it is possible to generate a unique list of 3D points and the inter-visibility of the points within the frames. Gordon and Lowe [3] mentioned that, in order to ensure the convergence of the BA, it is enough to place all the cameras at the same distance from the origin on the Z-axis and place all the projections in the XY-plane. It is important to highlight that it is necessary to keep track of the descriptors of the features, as they need to be stored with the 3D points as part of the model of the object. Figure 5 shows how the model has been iteratively constructed using the BA.

Moreover, before computing the BA, an additional filtering can be done to improve the performance. Therefore, due to the fact that the inter-visibility matrix was obtained, we can compute the number of times that each point appears (i.e., in how many images each point is observed). Some of the features can be badly matched or just not matched. Hence, this could produce duplicated points that might complicate the convergence of the algorithm. To avoid this, removing points that appear in less than *k* images can improve and speed up the convergence of the BA. The minimum value for *k* is 2, as the points need to appear in at least two images to be able to “triangulate” it. On the other hand, increasing this parameter too much is not possible, because the SBA solver might not be able to solve the problem if the number of observations is lower than the number of variables in the problem. Therefore, *k* is set to 3.

Once the BA process is performed, we obtain a 3D model of the object. However, as described in [4], because of the optimization algorithm, the points are not scaled according to the real size. Authors in [4] record an extra dataset in which the position and orientation of the object is known, then a second optimization algorithm is performed to obtain the correct model scale. In contrast, this extra dataset is not needed when using stereo cameras. As the correlation of all the points is known, it is possible to get the projections on both left and right images at each frame. $P^{m}$ are the points obtained from the BA, and $P^{t}$ is a cloud reconstructed from features of a frame *k* using the known stereo geometry. It is possible to estimate the transformation *T* between them using a SVD (Singular Value Decomposition) based estimator. *k* is the current frame, $N_{k}$ is the number of points seen on that frame, $p_{i}^{m}$ is the point *i* on the model, and $p_{i}^{t}$ is the triangulated point from the stereo pair; the score of each transformation is computed as

$$score=\sum_{i=1...N_{k}}(∥p_{i}^{m}-p_{i}^{t}∥)/N_{k}$$

the transformation that produces the minimum score is used to scale the model to the real-world size. Being

$$T=\left[\begin{array}{cccc} a_{11} & a_{12} & a_{13} & t_{x}\\ a_{21} & a_{22} & a_{23} & t_{y}\\ a_{31} & a_{32} & a_{33} & t_{z}\\ 0 & 0 & 0 & 1 \end{array}\right]$$

the scale factor can be computed as

$$s=\left[s_{x},s_{y},s_{z}\right]=\left\{\begin{array}{c} s_{x}=∥\left[a_{11},a_{21},a_{31}\right]∥\\ s_{y}=∥\left[a_{11},a_{21},a_{31}\right]∥\\ s_{z}=∥\left[a_{11},a_{21},a_{31}\right]∥ \end{array}\right.$$

Eventually, this model does not contain information about how to grasp it. This is done now manually in order to ensure a correct manipulation. As the object modeling is performed offline, it is realistic to choose it manually at this stage. Nevertheless, the detection of the grasping points can be analyzed depending on the manipulator and the geometry of the object using diverse quality metrics [2]—this is beyond the scope of this paper.

### 3.3. Finding Object in a Scene

In this subsection, the online detection of the object in new images and the position estimation is described. First of all, using the camera calibration, the acquired images are undistorted. The same feature detector and descriptor as the one used in the modeling stage is used to extract features in both input images. Then, the features in the pair of images are matched. As described before, the known parameters of the stereo calibration are used to filter the outliers. Hence, the remaining points are stronger as they appear in both cameras and they are easy to match.

At this point, there is a set of point candidates on the scene that become part of the object. To detect it and estimate its position, a PnP (Perspective-n-Points) formulation is used. $P=\{\left[x_{i},y_{i},z_{i}\right],\forall i=1...N\}$ is a set of 3D points, and $U=\{\left[u_{i},v_{i}\right],\forall i=1...N\}$ is their projection on the camera plane. The objective is to find the rotation *R* and translation *T* of the object in the camera’s coordinates (knowing the calibration parameters of the camera), minimizing the re-projection error of the points. Particularly, a RANSAC [39] implementation is used. It computes randomly possible solutions using the data matched between the scene and the model. Then, the matched points that lie far from the model are considered as outliers (i.e., rejected). In conclusion, it is less sensitive to local minima, and more robust to outliers.

**Figure 6:**
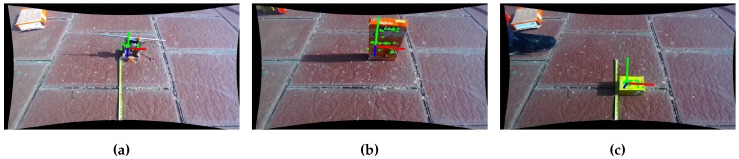
Examples of detection and position estimation of objects outdoors. White thin circles are candidate features in the scene. Green thick circles are the features assigned to the object, and the coordinate system is the representation of the position of the object. It depends on the coordinate system chosen at the modeling stage. (**a**) Drilling tool; (**b**) Whoopies box; (**c**) Gena box.

Now, we need to match the features in the scene with the model of the object to be able to start the PnP problem. In order to do that, each descriptor is matched with the points in the scene, and then filtered to remove outliers. The inliers are used in the PnP problem to detect the position of the object. Figure 6 shows screen-shots of results outdoors with a featured floor.

Nevertheless, during each step, several features belonging to the background are detected and described, which slows down the algorithm and increases the possibility of bad matches. In order to speed up the online stage, a moving window tracker was implemented. In the beginning, the algorithm searches for the objects over the whole image. However, if the confidence of the result is larger than a threshold, the expected portion of the next image in which the object appears is computed. As the pixel area of the following images are reduced, the amount of features computed decreases, and consequently, the algorithm runs faster. Algorithm 2 summarizes the process in the online stage.

**Algorithm 2** Online stage for finding learned objects.  1:searchWindow ← size(images)  2:**while** images ← camera **do**  3: Compute features on pair of images  4: Filter features  5: Match scene features with model features  6: Estimate object relative pose  7: **if** Number inliers threshold **then**  8:  Found object  9:  searchWindow ← boundBox(inliers)10:  move arm to relative pose11: **else**12:  searchWindow ← Size(images)13: **end** **if**14:**end** **while**



Finally, once the object is detected and its pose estimated, the algorithm sends the desired relative position to the robot arm and orients the gripper using the estimated orientation. This only happens if the number of detected inliers is higher than a manually set threshold. If the number of inliers decreased, the robot is returned to a safe position to start again when the object is detected again. Therefore, the gripper closes when the error between the estimated position of the object and the end-effector (using the direct-kinematic of the arm) is lower than a threshold. As mentioned before, the grasp position is determined in the offline stage, ensuring it is graspable for the designed arm.

## 4. Experimental Validation

### 4.1. Hardware Setup

In order to test the algorithm, a hexacopter was built and equipped with an on-board computer, the ZED cameras, and a 3DOF robotic arm (also designed by the authors). The UAV uses a F550 frame, and the engines were chosen to have a maximum thrust of 6 kg. The whole system (including batteries) weighs 4 kg. The arm is capable of lifting up to 500 g. The inertial measurement unit (IMU) and controller of the hexacopter is the well known 3DR PIXHAWK [40]. To ensure enough computational power, the computer that was used was an INTEL NUC5i7RYH [24]. This compact computer has a CPU i7 3.1 GHz and 8 GB of RAM. An Arduino Uno [41] board was added as an interface between the computer and the manipulator.

**Figure 7:**
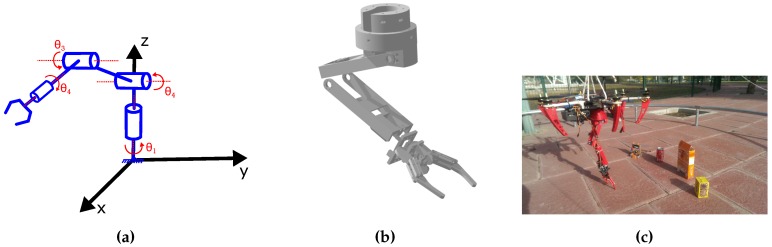
Model of robotic arm designed for the aerial robot. (**a**) Simplified model of the arm; (**b**) CAD design; (**c**) Final built-in platform.

The specifications of the robotic arm are: to be lightweight, have large range operation, and 3-DOF to accomplish the grasping task. Figure 7a shows a simplified model for the kinematics of the arm. Joints are represented in blue, and its variables in red. The inverse kinematic of the arm is governed by:

$$\left[\theta_{1},\theta_{2},\theta_{3}\right]=F\left(x,y,z\right)=\left\{\begin{array}{c} \theta_{1}=\mathrm{atan}\left(y/x\right)\\ \theta_{2}=\mathrm{acos}\left(\left(l_{2}^{2}-d^{2}-l_{1}^{2}\right)/\left(-2\times d\times l_{1}\right)\right)\\ \theta_{3}=\mathrm{acos}\left(\left(d^{2}-l_{1}^{2}-l_{2}^{2}\right)/\left(-2+l_{1}\times l_{2}\right)\right) \end{array}\right.$$

being, $d=\sqrt[{2}]{p^{2}+z^{2}}$ and $p=\sqrt[{2}]{x^{2}+y^{2}}$.

Figure 7b shows the CAD design of the parts, which were built by 3D printing. Finally, Figure 7c shows the whole structure that we built. The arm is attached to the bottom part of the drone (centered) with a custom piece that screws to the base of the robot and to the arm.

Additionally, a transformation is needed between the coordinate system of the camera and the coordinate system of the arm. This transformation is composed of a translation between the centers of the coordinates and a simple spin on the X axis:

$$T^{C\leftarrow A}=\left[\begin{array}{cccc} 1 & 0 & 0 & t_{x}\\ 0 & \cos(\alpha ) & -\sin(\alpha ) & t_{y}\\ 0 & \sin(\alpha ) & \cos(\alpha ) & t_{z}\\ 0 & 0 & 0 & 1 \end{array}\right]$$

The parameters of the transformation were experimentally obtained as α=30º, $t_{x}=0.06$ m, $t_{y}=0.1$ m, $t_{z}=0$.

### 4.2. Validation Tests

**Figure 8:**
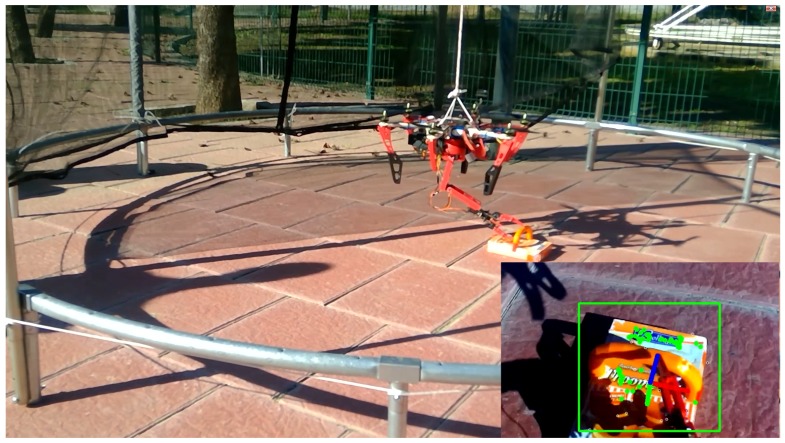
Robot grasping an object. The camera’s view is given in the inset. The green rectangle is the tracked moved window.

Figure 8 shows pictures of the testing environment. As mentioned, the UAV was hung on a structure for security reasons (since its control is not the target of this article). The objects were placed in its workspace so it could detect them and grasp them. The drone was controlled in loiter mode, describing up and down movements.

As mentioned in Section 3, the performance of the system depends on the election of the features extractor and descriptors. Table 1 summarizes the results for different combinations of detectors and descriptors at different resolutions in an outdoor environment. Results in indoor environments with poor light conditions are usually faster, generally because fewer features are detected so fewer features need to be described. Table 1 presents the performance of the algorithm for total image resolution without using the window tracker (i.e., before the object is tracked).

The algorithm’s execution time is divided mainly into two processes; the first is the feature detection and description (Table 1), matching, and filtering, and the second one is the PnP solving method. If the object is not on the scene, the PnP solver takes longer due to the fact that it does not converge, and it performs all the defined number of iterations. On the other hand, the time for the first stage is usually stable. This only depends on the choice of the detector and descriptor, and on the smoothness of the image.

The PnP process was analyzed regarding the confidence parameter and the reprojection error parameter. These two parameters affect the performance of the algorithm in both time and pose estimation. To give a numerical idea of the influence of the parameters, Table 2 summarizes the average time for the algorithm, varying the parameters using FAST and SIFT. The reprojection error is the maximum allowed “projection” error for the inliers, and the confidence parameter’s influence on the quality of the result.

**Figure 9:**
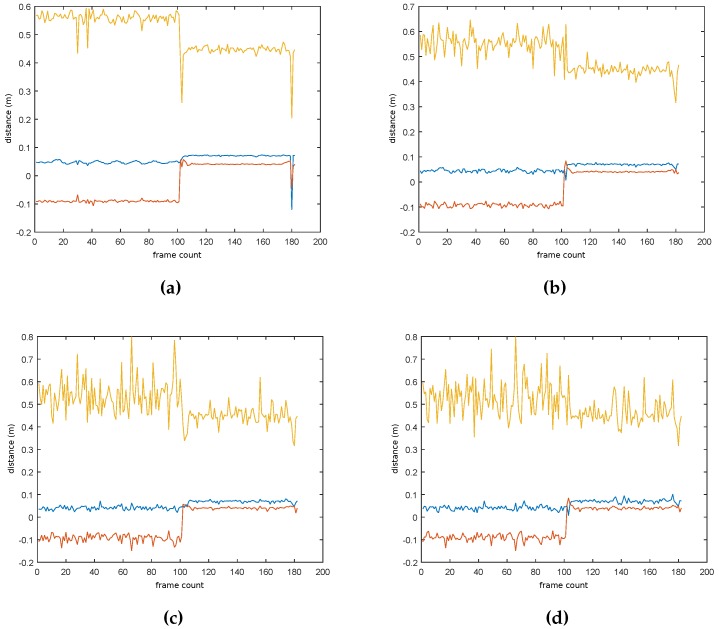
Result of pose estimation algorithm varying the reprojection error. Lines from top to bottom are: Z-coordinate (yellow), X-coordinate (blue), and Y-coordinate (red). Increasing the parameter decreases the quality of the results. However, as described in Table 2, it is slightly faster. (**a**) Reprojection error 3; (**b**) Reprojection error 5; (**c**) Reprojection error 7; (**d**) Reprojection error 8.

Increasing the reprojection error increases the speed, but as shown in Figure 9, results in worsening of the position. Similarly, decreasing the confidence parameter speeds up the PnP algorithm, but decreases the quality of the result. Figure 9 shows the estimated position of an object, varying the reprojection parameter. It can be seen that the estimation on the Z axis (forward direction of the camera) is worst when the reprojection error increases. As the projections of the points are allowed to fall further, larger errors in translation and rotation can be produced. Additionally, these computation times are reduced up to 75% thanks to the optimization described in Algorithm 2.

**Figure 10:**
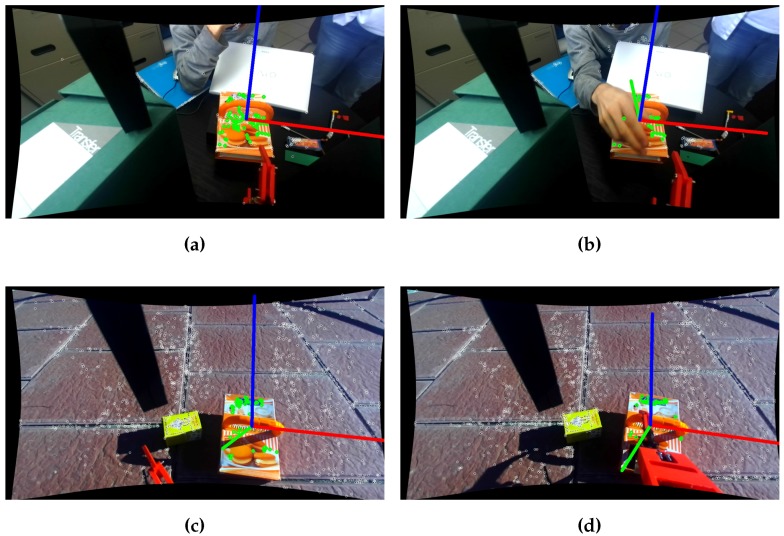
Testing detection and position estimation with partial occlusions. (**a**) Non-occluded indoor; (**b**) Occluded indoor; (**c**) Non-occluded outdoor; (**d**) Occluded outdoor. The top figures show an indoor test, where the object is occluded by a human hand. In the bottom figures, the object is occluded by the arm of the UAV during the grasp process.

The algorithm is also proven to be robust to occlusions. The position of the object can be reconstructed with a small fraction of points of the model. Figure 10a,b shows the estimated position of an object occluded partially by a person. Additionally, in Figure 10c,d, one can see how the position of the object remains stable, even with the arm occluding the object during the grasping trajectory. It is noticeable that the position is more stable than the orientation against partial occlusions.

**Figure 11:**
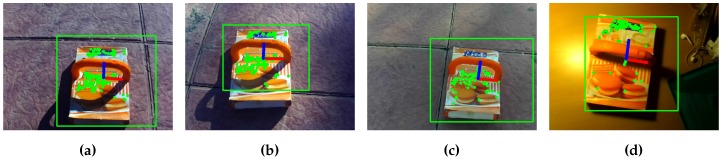
Testing algorithm with different light conditions. (**a**) Without shadow; (**b**) Partial tree shadow; (**c**) Complete tree shadow; (**d**) Lamp light.

The performance of the FAST/SIFT features under different light conditions was also validated, as can be seen in Figure 11. This figure shows the result of the object detection and pose estimation with different types of lights and shadows (indoor, outdoor with shadows, and without shadows). The algorithm performs well in outdoor environments with and without shadows, as the camera automatically adjusts the exposure of the sensor. Indoors, the result is initially the same. However, as the exposure time of the camera increases, the image is more prone to have motion blur. This implies large variations in the feature descriptors. Because of this, the algorithm may lose the object tracking during fast movements.

It was observed that the use of the FAST detector and SIFT descriptors produced the best results. In the learning stage, these features produced accurate models. Subsequently, the position estimation was recovered—in both indoor and outdoor environments—more easily than with the other feature descriptors. However, the computation time of this descriptor is too high. Indoors, a frame speed within 8 and 13 FPS was obtained without using Algorithm 2, and 15–19 FPS using it. Outdoors, however, due to the light conditions and the texture of the floor, the FPS decreased drastically to within 2 and 4 FPS without optimization and up to 11 FPS with the moving window. This happened because the feature detector detects more features outdoors, as images are sharper.

The second-best option is the use of the FAST detector and rBRIEF (rotated BRIEF). The computation time for this descriptor is significantly lower than SIFT, and the computation of distances in the PnP solver takes less time, as the descriptors are smaller. It works within 25–30 FPS indoors and reaches 25 outdoors thanks to the optimization. Nevertheless, this descriptor showed the worst behavior against variations in the scale.

**Figure 12:**
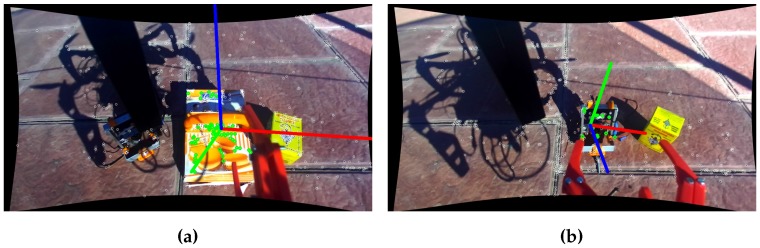
Testing grasp with multiple objects. The algorithm is able to switch the targeted object according to any desired task if the model is learned. (**a**) Picking whoopies box; (**b**) Picking drilling tool.

Last but not least, the algorithm was tested with multiple objects at the same time in the scene. Figure 12 shows the results obtained by varying the chosen object model for the detection.

## 5. Conclusions and Future Work

An on-board object detection method for an aerial robot that computes the needed information for the autonomous grasping of objects was developed. The algorithm was tested outdoors to test strong light conditions and its robustness against the vibrations generated by the UAV. The UAV was provided with a lightweight 3DOF arm for proof of concept of grasping objects.

In contrast to previous work, stereo cameras were chosen for two reasons: (1) to automate the learning process (the images are filtered using the stereo geometry, and the scale of the object is obtained automatically from the set of images without needing a manually calibrated dataset); and (2) for filtering bad features in the detection stage, making it more robust.

This can be used in several manipulation applications, such as inspection or maintenance of pipes or wind turbines. A drop-off/pick-up zone for objects (sensors, tools, etc.) can be selected, and the drone is able to pick up objects autonomously without requiring any information about the exact location. In contrast to RGB-D systems, the proposed method can be used robustly in outdoor environments. Furthermore, the method performs well under occlusions and the presence of outliers due to the feature-based modeling of the objects.

A speed comparison of different features has been made. This made it possible to choose the features that are better suited to the problem. As mentioned, the SIFT descriptors are more robust, as they perform well with different rotations and scales. However, this descriptor is slower than others, so if the UAV needs faster results, it is better to use other descriptors. rBRIEF (rotated BRIEF) is a good alternative. It is much faster than SIFT, and it is also invariant to rotations. Its main disadvantage is being less robust to scales.

As a future step, it might be interesting to compute the grasping points using quality metrics instead of choosing them manually at the learning stage. So far, all the tests have been performed by tying the UAV to a secure structure. The next step is to perform experiments while undertaking an autonomous flight. Finally, we want to speed up the feature detection using GPU to reduce the CPU computations and allow the UAV to perform more operations on the computer.

## Figures and Tables

**Table 1:** Average computational times for the feature detection, matching, and stereo filtering for different feature detectors and descriptors. Using different image resolutions (640 × 480 and 1280 × 720). FAST: features from accelerated segment test; SIFT: scale-invariant feature transform; SURF: speeded up robust features; BRIEF: binary robust independent elementary features; rBRIEF: rotated BRIEF; DAISY: an efficient dense descriptor applied for wide baseline stereo.

	FAST Detector		SIFT Detector		SURF Detector	
	640 × 480	1280 × 720	640 × 480	1280 × 720	640 × 480	1280 × 720
SIFT descriptor	0.318 s	0.739 s	0.510 s	1.299 s	1.532 s	2.412 s
BRIEF descriptor	0.042 s	0.214 s	0.250 s	0.660 s	0.235 s	1.012 s
rBRIEF descriptor	0.045 s	0.229 s	0.237 s	0.715 s	0.256 s	1.100 s
SURF descriptor	0.074 s	0.215 s	0.380 s	0.986 s	0.368 s	1.098 s
DAISY descriptor	0.319 s	0.876 s	0.523 s	1.516 s	0.489 s	1.421 s

**Table 2:** Computation times of the PnP (Perspective-n-Points) algorithm varying the confidence parameter and the reprojection error.

	Reprojection Error			
	3 pxs.	5 pxs.	7 pxs.	8 pxs.
confidence=0.99	0.031 s	0.028 s	0.025 s	0.024 s
confidence=0.999	0.036 s	0.031 s	0.027 s	0.026 s
confidence=0.9999	0.039 s	0.034 s	0.028 s	0.028 s
